# Combining CEUS and CT/MRI LI-RADS major imaging features: diagnostic accuracy for classification of indeterminate liver observations in patients at risk for HCC

**DOI:** 10.1007/s00261-024-04625-w

**Published:** 2024-10-23

**Authors:** Tania Siu Xiao, Cristina Mariuxi Kuon Yeng Escalante, Aylin Tahmasebi, Yuko Kono, Fabio Piscaglia, Stephanie R. Wilson, Alexandra Medellin-Kowalewski, Shuchi K. Rodgers, Virginia Planz, Aya Kamaya, David T. Fetzer, Annalisa Berzigotti, Iuliana-Pompilia Radu, Paul S. Sidhu, Corinne E. Wessner, Kristen Bradigan, John R. Eisenbrey, Flemming Forsberg, Andrej Lyshchik

**Affiliations:** 1 Department of Radiology, Thomas Jefferson University Hospital, Philadelphia, USA; 2 Division of Gastroenterology and Hepatology, Department of Medicine, University of California, San Diego, USA; 3 Division of Internal Medicine, Hepatobiliary and Immunoallergic Diseases, IRCCS Azienda Ospedaliero-Universitaria di Bologna, Bologna, Italy; 4 Department of Medical and Surgical Sciences, University of Bologna, Bologna, Italy; 5 Department of Radiology, University of Calgary, Calgary, Canada; 6 Department of Radiology, Einstein Medical Center Philadelphia, Philadelphia, USA; 7 Department of Radiology, Vanderbilt University, Nashville, USA; 8 Department of Radiology, Stanford University, Stanford, USA; 9 Department of Radiology, UT Southwestern Medical Center, Dallas, USA; 10 Inselspital, Bern University Hospital, University of Bern, Bern, Switzerland; 11 Department of Radiology, King’s College Hospital, London, UK

**Keywords:** CEUS, LI-RADS, CT, MRI, HCC, Hepatocellular carcinoma

## Abstract

**Purpose:**

To determine the diagnostic accuracy of combining CEUS and CT/MRI LI-RADS major imaging features for the improved categorization of liver observations indeterminate on both CT/MRI and CEUS.

**Materials and methods:**

A retrospective analysis using a database from a prospective study conducted at 11 centers in North America and Europe from 2018 to 2022 included a total of 109 participants at risk for HCC who had liver observations with indeterminate characterization (LR3, LR-4, and LR-M) on both CEUS and CT/MRI. The individual CEUS and CT/MRI LI-RADS major features were extracted from the original study and analyzed in various combinations. Reference standards included biopsy, explant histology, and follow-up CT/MRI. The diagnostic performance of the combinations of LI-RADS major features for definitive diagnosis of HCC was calculated. A reverse, stepwise logistical regression sub-analysis was also performed.

**Results:**

This study included 114 observations indeterminate on both CT/MRI and CEUS. These observations were categorized as LR-3 (n = 37), LR-4 (n = 41), and LR-M (n = 36) on CT/MRI and LR-3 (n = 48), LR-4 (n = 36), LR-M (n = 29), and LR-TIV (n = 1) on CEUS. Of them, 43.0% (49/114) were confirmed as HCC, 37.3% (43/114) non-malignant, and 19.3% (22/114) non-hepatocellular malignancies. The highest diagnostic accuracy among the combinations of imaging features was achieved in CT/MRI LR-3 observations, where the combination of CEUS arterial phase hyper-enhancement (APHE) + CT/MRI APHE had 96.7% specificity, 75.0% positive predictive value (PPV), and 86.5% accuracy for HCC.

**Conclusion:**

The combination of LI-RADS major features on CT/MRI and CEUS showed higher specificity, PPV, and accuracy compared to individual modalities' assessments, particularly for CT/MRI LR-3 observations.

**Supplementary Information:**

The online version contains supplementary material available at 10.1007/s00261-024-04625-w.

## Introduction

According to the American Cancer Society, the sixth most common cancer and the third greatest cause of death worldwide is primary liver cancer [1]. The most common being hepatocellular carcinoma (HCC), comprising more than 80% of cases [2]. Unfortunately, the prognosis is poor, with a 5-year survival rate of less than 20%, making early detection and definitive diagnosis a priority [3]. Additionally, diagnosing HCC in early stages is associated with improved outcomes [4, 5].

The American College of Radiology Liver Imaging Reporting and Data System (LI-RADS) is a comprehensive system with algorithms describing the use of contrast-enhanced ultrasound (CEUS) and CT/MRI to categorize observations in patients at risk for HCC [6, 7]. CEUS LI-RADS v2017 and CT/MRI LI-RADS v2018 describe major features for HCC, including arterial phase hyper-enhancement (APHE), tumor size, washout (WO), capsule enhancement, and threshold growth [6]. Timing and degree of WO with CEUS are important, with early or marked WO used as major features for LR-M (malignant, but not HCC specific), and late and mild WO as a requirement for LR-5 (definitely HCC) categorization [8]. Capsule enhancement on CT/MRI has a high specificity in diagnosing HCC [9, 10].

According to the current clinical standards of care, the definitive diagnosis of HCC can be established with noninvasive imaging techniques without the need for biopsy [2, 11, 12]. LI-RADS has been widely adopted for this purpose. Unfortunately, close to 50% of liver observations in patients at risk fall into indeterminate categories for HCC, such as LR-3 (intermediate probability for HCC), LR-4 (probably HCC), or LR-M [13, 14]. The rate of HCC within these three categories spans from approximately 37% to 82% [11]. The American Association for the Study of Liver Diseases guidelines recommend ongoing surveillance with repeat CT or MRI every three to six months for LR-3 observations, multidisciplinary discussion on follow-up imaging at 3 months, percutaneous biopsy, or treatment for LR-4 observations, and biopsy for LR-M observations [2]. Nevertheless, surveillance approaches for LR-3 and LR-4 carry the risk of progression to more advanced tumor conditions with fewer options for effective treatment, compared to a more desirable accurate categorization from the outset [15]. Therefore, developing new strategies to improve the initial categorization of indeterminate liver observations as HCC is of extreme importance.

This study aims to determine whether combining the major features of CEUS and CT/MRI LI-RADS in observations with indeterminate categorization on both CT/MRI and CEUS could improve the definitive diagnosis of HCC.

## Methods

### Study design and participants

**Fig. 1:**
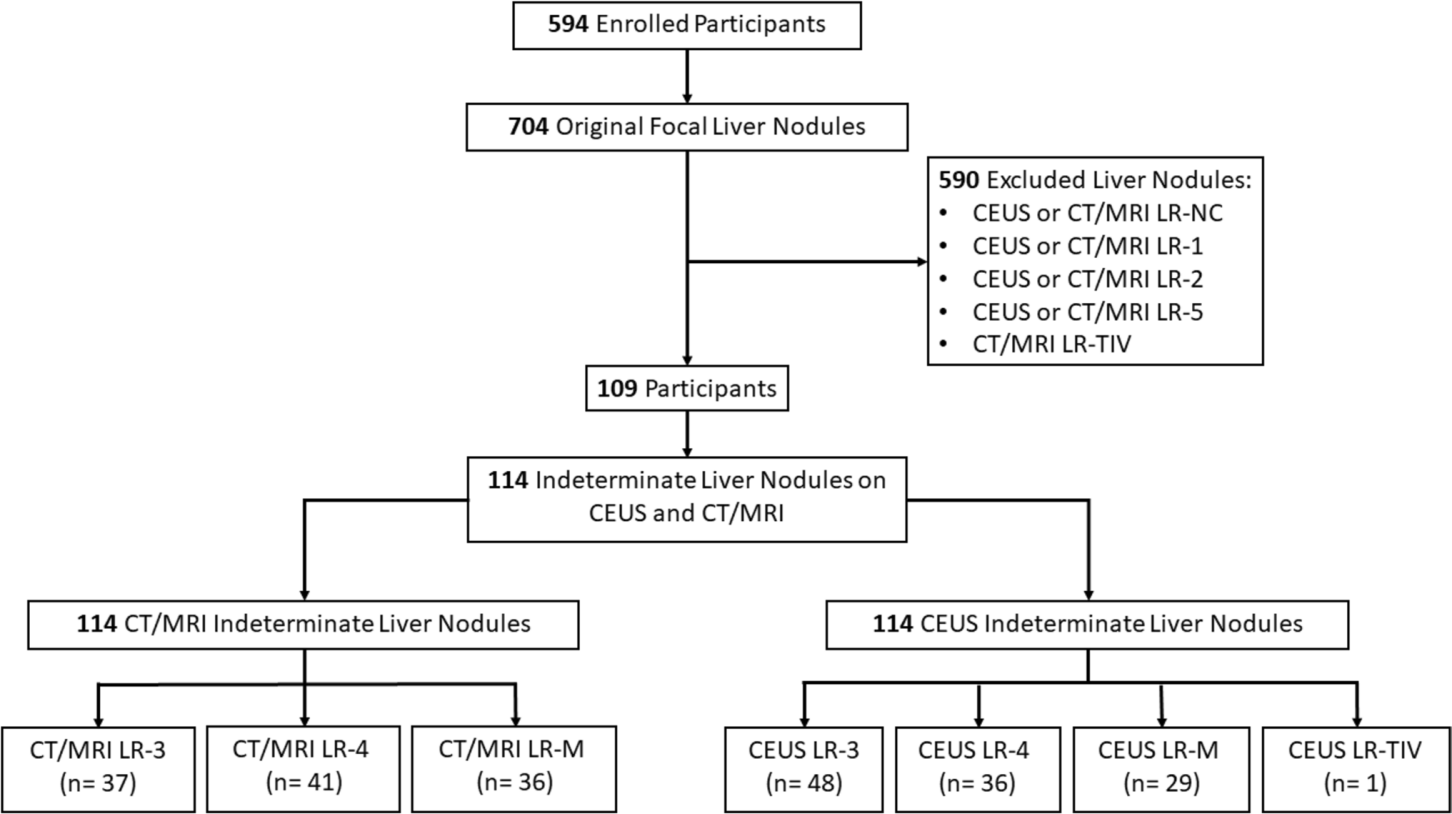
Study flowchart**.** *CEUS* contrast-enhanced ultrasound, *CT* computed tomography, *MRI* magnetic resonance imaging

This is a retrospective analysis using a database from a recently completed prospective study that included 11 academic and nonacademic centers in North America and Europe conducted between 2018 and 2022, which evaluated the diagnostic performance of CEUS in patients at risk of HCC [11]. All patients provided consent covering the initial study and subsequent data analyses. The race, ethnicity, and clinical data were collected from the electronic medical records. The study flowchart is shown in Fig. 1. Originally, 594 participants were enrolled in the previous study, from which 704 liver observations were identified [11]. Of them, only indeterminate liver observations (LR-3, LR-4, and LR-M) by both CEUS and CT/MRI were included in the analysis, resulting in a total of 114 liver observations. CEUS or CT/MRI LR-NC, LR-1, LR-2, LR-5, and CT/MRI LR-TIV liver observations were excluded, resulting in 590 excluded observations. The previous study evaluated the diagnostic accuracy of CEUS LI-RADS LR-5 characterization for diagnosing HCC [11]. The contrast agents used were LUMASON® (sulfur hexafluoride lipid-type A microspheres) and SonoVue® (sulfur hexafluoride) from Bracco Diagnostics, Monroe Township, NJ/Bracco Spa, Milan, Italy. In contrast, the current study analyzes the outcome of combining CEUS and CT/MRI LI-RADS major features in indeterminate liver observations to determine the accuracy of HCC diagnosis.

Individual LI-RADS major features (presence of APHE, WO, capsule enhancement, and threshold growth for CT/MRI; and APHE, late and mild WO for CEUS) of each patient were extracted from the original study. First, the diagnostic performance of each imaging feature alone was quantified. Second, the cases were divided into LR-3, LR-4, and LR-M according to the initial CT/MRI LI-RADS classification. Finally, the different inter-modality LI-RADS major feature combinations were grouped as shown in Online Resource 1, and the analyses of these combinations were performed on each category of indeterminate liver observation based on initial CT/MRI LI-RADS classification (CT/MRI LR-3, LR-4, and LR-M).

A sub-analysis using a reverse, stepwise logistical regression was used to determine which variables (if any) contribute to the definitive diagnosis of HCC (LR-5). Area under the receiver operating characteristic curve (A_z_) with statistical significance *P*-value of 0.05 or lower was selected as statistical significance level. The variables used in the analysis are summarized in Online Resource 2. Variables were assessed with pathology as the outcome (i.e., the dependent variable). Results were compared for benign versus all malignant observations as subgroup 1 and HCC versus everything else (benign and non-HCC malignancy) as subgroup 2.

### Reference standard

A composite reference standard based on either biopsy, explant histology, or follow-up CT/MRI was used. Instead of each patient, each liver observation was used as the unit of analysis. When available, histopathology was used for all observations, regardless of their initial imaging characterization. For observations initially classified as CT/MRI LR-M, histological confirmation was required, and only cases with this confirmation were included. For observations initially classified as CT/MRI LR-3 and LR-4 and without histopathological confirmation, follow-up CT/MRI imaging was performed.

### Statistical analyses

The statistical analyses were performed according to their initial CT/MRI LI-RADS classification, as LR-3 alone, LR-4 alone, LR-M alone, LR-3 + LR4, and all indeterminate liver observations (LR-3, LR-4, and LR-M). To determine the diagnostic efficacy of separated and combined imaging modalities (CEUS and CT/MRI) using LI-RADS criteria for the diagnosis of HCC in participants with initial CEUS and CT/MRI LR-3, LR-4, and LR-M, we assessed their sensitivity, specificity, positive predictive value (PPV), negative predictive value (NPV), and accuracy. The statistical analyses were calculated using commercially available MedCalc Software for Windows (v22.019—Jan 29, 2024; MedCalc Software, Ostend, Belgium) [16].

The reverse, stepwise logistical regression was performed using Stata version 15.1 (Stata Corp LLC, College Station, TX).

## Results

### Participants

Based on the selection criteria, 109 participants with 114 indeterminate liver observations based on CEUS and CT/MRI were included in the study (Fig. 1). Two liver observations were present in five participants. The participant demographics and important clinical information are summarized in Table 1. Mean (± standard deviation) age was 61.6 years ± 10.5 years, and 78/109 (71.6%) of the participants were male.

**Table 1:** Participant demographics and important clinical information

*Parameter*	*No. of Participants (%)*
*Gender*	
*Female*	31 (28.4%)
*Male*	78 (71.6)
Age (Years)*	61.6 ± 10.5 (22–85)*
*Race*	
*Asian*	3 (2.8)
*African American*	6 (5.5)
*Native Hawaiian or Other Pacific Islander*	1 (0.9)
*White*	79 (72.5)
*Unknown*	20 (18.3)
*Ethnicity*	
*Hispanic or Latino*	21 (19.3)
*Not Hispanic or Latino*	86 (78.9)
*Unknown*	2 (1.8)
*Body mass index (kg/m*^*2*^)*	29.1 ± 5.3 (18.8–45.3)*
*Liver Disease Etiology*	
*Alcohol*	41 (30.8)
*MASH*	27 (20.3)
*Hepatitis B*	11 (8.3)
*Hepatitis C*	44 (33.1)
*Other*	10 (7.5)
*Cirrhosis*	
*Yes*	105 (96.3)
*No*	4 (3.7)
*Encephalopathy*	
*No encephalopathy*	95 (87.1)
*Grade 1–2*	10 (9.2)
*Grade 3–4*	0 (0.0)
*Unknown*	4 (3.7)
*Ascites*	
*Absent*	74 (67.9)
*Slight*	23 (21.1)
*Moderate*	8 (7.3)
*Unknown*	4 (3.7)
*Child–Pugh Classification*	
*A*	70 (64.2)
*B*	27 (24.8)
*C*	8 (7.3)
*Unknown*	4 (3.7)

Unless otherwise indicated, data are numbers of participants with percentages in parentheses

*Data are means ± SDs and data in parentheses are ranges

*HCC* Hepatocellular carcinoma, *MASH* Metabolic dysfunction-associated steatohepatitis, *SD* Standard deviation

### Distribution of liver observations and reference standard

**Fig. 2:**
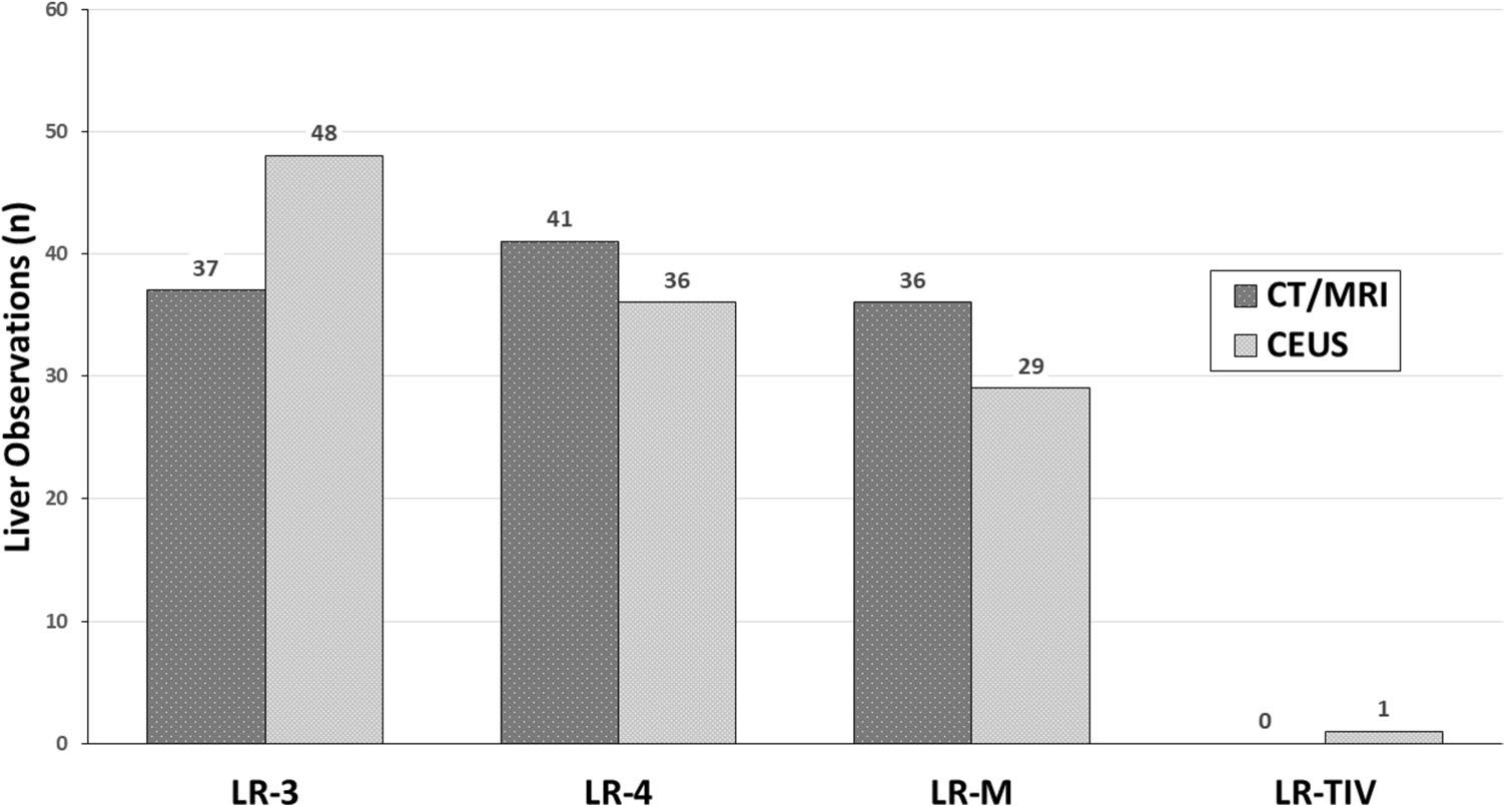
Distribution of total initially indeterminate liver observations on CT/MRI and CEUS. *CEUS* contrast-enhanced ultrasound, *CT* computed tomography, *MRI* magnetic resonance imaging

The distribution of liver observations on CEUS and CT/MRI are shown in Fig. 2. Based on CT/MRI LI-RADS, liver observations were categorized as LR-3 in 32.4% (37/114), LR-4 in 36.0% (41/114), and LR-M in 31.6% (36/114). Liver observations by CEUS LI-RADS were categorized as LR-3 in 42.1% (48/114), LR-4 in 31.6% (36/114), LR-M in 25.4% (29/114), and LR-TIV in 0.9% (1/114).

**Fig. 3:**
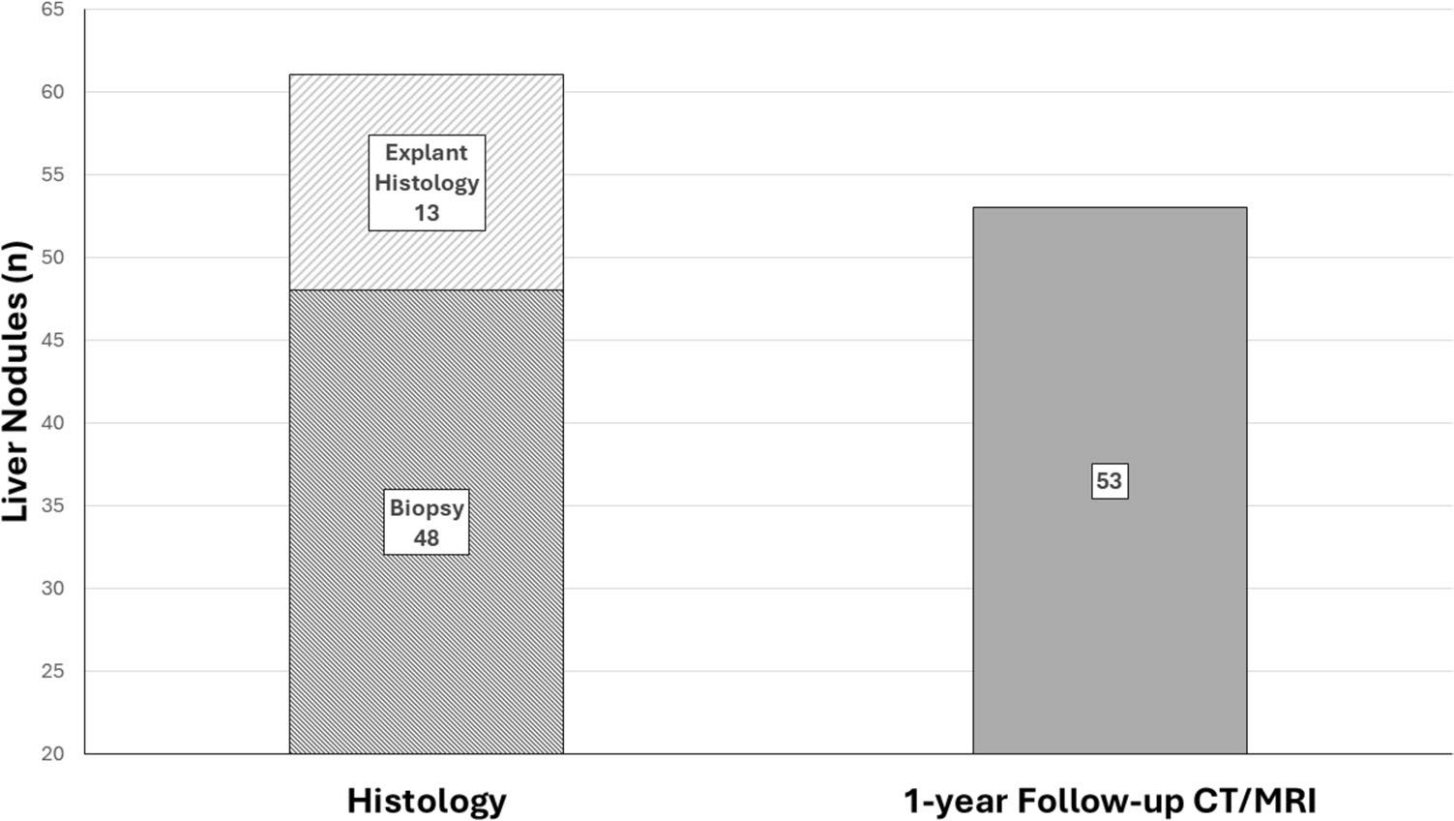
Final diagnosis source of initially indeterminate liver observations. *CT* computed tomography, *MRI* magnetic resonance imaging

According to the reference standard, 43.0% (49/114) were confirmed as HCC, 37.3% (43/114) were benign, and 19.3% (22/114) were non-HCC malignancies (Online Resource 3). By the initial CT/MRI classification, LR-3 (n = 37), 78.4% (29/37) were confirmed as benign, 18.9% (7/37) were HCC, and 2.7% (1/37) was non-HCC malignancy; for LR-4 (n = 41), 31.7% (13/41) were benign, 56.1% (23/41) were HCC, and 12.2% (5/41) were non-HCC malignancies; and for LR-M (n = 36), 2.8% (1/36) was benign, 52.8% (19/36) were HCC, and 44.4% (16/36) were non-HCC malignancies (Online Resource 3). Among all the non-HCC malignancies (22/114), 12 were confirmed intrahepatic cholangiocarcinoma, 3 combined hepatocellular carcinoma-cholangiocarcinoma, 4 adenocarcinoma, 1 colon carcinoma metastasis, 1 metastatic carcinoma likely of pancreatic or gastrointestinal origin, and 1 poorly differentiated carcinoma. The source of the final diagnosis was based on histology in 53.5% (61/114), of which 48 were biopsy and 13 explant histology, and 1-year follow-up CT/MRI in 46.5% (53/114) (Fig. 3). A direct comparison of initial CEUS and CT/MRI LI-RADS classification (Online Resource 4) with final diagnosis and CEUS and CT/MRI LI-RADS major features (Online Resource 5) was performed.

### Distribution of CEUS and CT/MRI LI-RADS major features

Of the 114 observations, APHE was observed in 34% (39/114) with CEUS and 46% (52/114) with CT/MRI. WO was seen in 36% (41/114) and 27% (31/114) with CEUS and CT/MRI, respectively. For CEUS, late and mild WO was reported in 20% (23/114) and early or marked WO in 15.8% (18/114) of liver observations. For CT/MRI, capsule enhancement and threshold growth were seen in 8% (9/114) and 13% (15/114) of liver observations, respectively.

### Diagnostic accuracy of individual LI-RADS major features in CEUS and CT/MRI

The sensitivity, specificity, PPV, NPV, and accuracy for each individual LI-RADS major feature for CEUS and CT/MRI across all indeterminate liver observations (CT/MRI LR-3, LR-4, and LR-M) are shown in Table 2. The analysis showed sensitivities of 46.9%, 51.0%, 30.6%, 32.7%, 20.4%, 12.2%, and 12.2% for CEUS APHE, CT/MRI APHE, CEUS WO, CT/MRI WO, CEUS late and mild WO, CT/MRI capsule enhancement, and CT/MRI threshold growth, whereas the specificities were 75.4%, 58.5%, 60.0%, 76.9%, 80.0%, 95.4%, and 86.2%, respectively. The PPV ranged from 36 to 66% for individual modalities.

**Table 2:** Individual LI-RADS major features by modality across all indeterminate liver observations

*Modality with LI-RADS major features*	Sensitivity	Specificity	PPV	NPV	Accuracy
*CEUS APHE*	23/49 (46.9)	49/65 (75.4)	23/39 (59.0)	49/75 (65.3)	72/114 (63.2)
*CT/MRI APHE*	25/49 (51.0)	38/65 (58.5)	25/52 (48.1)	38/62 (61.3)	63/114 (55.3)
*CEUS WO*	15/49 (30.6)	39/65 (60.0)	15/41 (36.6)	36/73 (53.4)	54/114 (47.4)
*CT/MRI WO*	16/49 (32.7)	50/65 (76.9)	16/31 (51.6)	50/83 (60.2)	66/114 (57.9)
*CEUS late and mild WO*	10/49 (20.4)	52/65 (80.0)	10/23 (43.5)	52/91 (57.1)	62/114 (54.4)
*CT/MRI capsule enhancement*	6/49 (12.2)	62/65 (95.4)	6/9 (66.7)	62/105 (59.1)	68/114 (59.7)
*CT/MRI threshold growth*	6/49 (12.2)	56/65 (86.2)	6/15 (40.0)	56/99 (56.6)	62/114 (54.4)

Data are numerators/denominators. Data in parenthesis are percentages

*APHE* Arterial-phase hyper-enhancement, *CEUS* Contrast-enhanced ultrasound, *CT* Computed tomography, *LI-RADS* Liver imaging reporting and data system, *MRI* Magnetic resonance imaging, *NPV* Negative predictive value, *PPV* Positive predictive value, *WO* Washout (regardless of timing or degree)

### Diagnostic accuracy of combined CEUS and CT/MRI LI-RADS major features

The sensitivity, specificity, PPV, NPV, and accuracy for each combined LI-RADS major features from CEUS and CT/MRI across all indeterminate liver observations (CT/MRI LR-3, LR-4, and LR-M) and CT/MRI LR-M alone, and CT/MRI LR-3 alone, CT/MRI LR-4 alone, and CT/MRI LR-3 + LR4 are summarized in Tables 3 and 4, respectively.

**Table 3:** Multimodality imaging with LI-RADS major features across all indeterminate liver observations and CT/MRI LR-M alone

GROUPS	Sensitivity	Specificity	PPV	NPV	Accuracy
*All indeterminate observations*					
*1. CEUS APHE* + *CT/MRI APHE*	14/49 (28.6)	55/65 (84.6)	14/24 (58.3)	55/90 (61.1)	69/114 (60.5)
*2. CEUS APHE* + *CT/MRI WO*	7/49 (14.3)	62/65 (95.4)	7/10 (70.0)	62/104 (59.6)	69/114 (60.5)
*3. CEUS WO* + *CT/MRI APHE*	4/49 (8.2)	57/65 (87.7)	4/12 (33.3)	57/102 (55.9)	61/114 (53.5)
*4. CEUS WO* + *CT/MRI WO*	6/49 (12.2)	60/65 (92.3)	6/11 (54.6)	60/103 (58.3)	66/114 (57.9)
*5. CT/MRI APHE* + *CEUS late and mild WO*	3/49 (6.1)	62/65 (95.4)	3/6 (50.0)	62/108 (57.4)	65/114 (57.0)
*6. CT/MRI WO* + *CEUS late and mild WO*	5/49 (10.2)	63/65 (96.9)	5/7 (71.4)	63/107 (58.9)	68/114 (59.7)
*7. CEUS APHE* + *CT/MRI (capsule enhancement)*	3/49 (6.1)	64/65 (98.5)	3/4 (75.0)	64/110 (58.2)	67/114 (58.8)
*8. CEUS APHE* + *CT/MRI (threshold growth)*	2/49 (4.1)	63/65 (96.9)	2/4 (50.0)	63/110 (57.3)	65/114 (57.0)
*9. CEUS late and mild WO* + *CT/MRI (capsule enhancement)*	1/49 (2.0)	65/65 (100)	1/1 (100)	65/113 (57.5)	66/114 (57.9)
*10. CEUS late and mild WO* + *CT/MRI (threshold growth)*	4/49 (8.2)	628/65 (95.4)	4/7 (57.1)	62/107 (57.9)	66/114 (57.9)
*CT/MRI LR-M alone*					
*1. CEUS APHE* + *CT/MRI APHE*	3/19 (15.8)	14/17 (82.4)	3/6 (50.0)	14/30 (46.7)	17/36 (47.2)
*2. CEUS APHE* + *CT/MRI WO*	3/19 (15.8)	15/17 (88.2)	3/5 (60.0)	15/31 (48.4)	18/36 (50.0)
*3. CEUS WO* + *CT/MRI APHE*	1/19 (5.3)	14/17 (82.4)	1/4 (25.0)	14/32 (43.8)	15/36 (41.7)
*4. CEUS WO* + *CT/MRI WO*	3/19 (15.8)	13/17 (76.5)	3/7 (42.9)	13/29 (44.8)	16/36 (44.4)
*5. CT/MRI APHE* + *CEUS late and mild WO*	0/19 (0)	17/17 (100)	0/0 (0)	17/36 (47.2)	17/36 (47.2)
*6. CT/MRI WO* + *CEUS late and mild WO*	2/19 (10.5)	16/17 (94.1)	2/3 (66.7)	16/33 (48.5)	18/36 (50.0)
*7. CEUS APHE* + *CT/MRI (capsule enhancement)*	0/19 (0)	17/17 (100)	0/0 (0)	17/36 (47.2)	17/36 (47.2)
*8. CEUS APHE* + *CT/MRI (threshold growth)*	1/19 (5.3)	16/17 (94.1)	1/2 (50.0)	16/34 (47.1)	17/36 (47.2)
*9. CEUS late and mild WO* + *CT/MRI (capsule enhancement)*	0/19 (0)	17/17 (100)	0/0 (0)	17/36 (47.2)	17/36 (47.2)
*10. CEUS late and mild WO* + *CT/MRI (threshold growth)*	3/19 (15.8)	16/17 (94.1)	3/4 (75.0)	16/32 (50.0)	19/36 (52.8)

Data are numerators/denominators. Data in parenthesis are percentages

*APHE* Arterial-phase hyper-enhancement, *CEUS* Contrast-enhanced ultrasound, *CT* Computed tomography, *LI-RADS* Liver Imaging reporting and data system, *MRI* Magnetic resonance imaging, *NPV* Negative predictive value, *PPV* Positive predictive value, *WO* Washout (regardless of timing or degree)

**Table 4:** Multimodality imaging with LI-RADS major features in CT/MRI LR-3 alone, CT/MRI LR-4 alone, and CT/MRI LR-3 + LR-4

GROUPS	Sensitivity	Specificity	PPV	NPV	Accuracy
*CT/MRI LR-3 alone*					
*1. CEUS APHE* + *CT/MRI APHE*	3/7 (42.9)	29/30 (96.7)	3/4 (75.0)	29/33 (87.9)	32/37 (86.5)
*2. CEUS APHE* + *CT/MRI WO*	1/7 (14.3)	29/30 (96.7)	1/2 (50.0)	29/35 (82.9)	30/37 (81.1)
*3. CEUS WO* + *CT/MRI APHE*	0/7 (0)	28/30 (93.3)	0/2 (0)	28/35 (80.0)	28/37 (75.7)
*4. CEUS WO* + *CT/MRI WO*	0/7 (0)	30/30 (100)	0/0 (0)	30/37 (81.1)	30/37 (81.1)
*5. CT/MRI APHE* + *CEUS late and mild WO*	0/7 (0)	28/30 (93.3)	0/2 (0)	28/35 (80.0)	28/37 (75.7)
*6. CT/MRI WO* + *CEUS late and mild WO*	0/7 (0)	30/30 (100)	0/0 (0)	30/37 (81.1)	30/37 (81.1)
*7. CEUS APHE* + *CT/MRI (capsule enhancement)*	0/7 (0)	30/30 (100)	0/0 (0)	30/37 (81.1)	30/37 (81.1)
*8. CEUS APHE* + *CT/MRI (threshold growth)*	0/7 (0)	30/30 (100)	0/0 (0)	30/37 (81.1)	30/37 (81.1)
*9. CEUS late and mild WO* + *CT/MRI (capsule enhancement)*	0/7 (0)	30/30 (100)	0/0 (0)	30/37 (81.1)	30/37 (81.1)
*10. CEUS late and mild WO* + *CT/MRI (threshold growth)*	0/7 (0)	30/30 (100)	0/0 (0)	30/37 (81.1)	30/37 (81.1)
*CT/MRI LR-4 alone*					
*1. CEUS APHE* + *CT/MRI APHE*	8/23 (34.8)	12/18 (66.7)	8/14 (57.1)	12/27 (44.4)	20/41 (48.8)
*2. CEUS APHE* + *CT/MRI WO*	3/23 (13.0)	18/18 (100)	3/3 (100)	18/38 (47.4)	21/41 (51.2)
*3. CEUS WO* + *CT/MRI APHE*	3/23 (13.0)	15/18 (83.3)	3/6 (50.0)	15/35 (42.9)	18/41 (43.9)
*4. CEUS WO* + *CT/MRI WO*	3/23 (13.0)	17/18 (94.4)	3/4 (75.0)	17/37 (46.0)	20/41 (48.8)
*5. CT/MRI APHE* + *CEUS late and mild WO*	3/23 (13.0)	17/18 (94.4)	3/4 (75.0)	17/37 (46.0)	20/41 (48.8)
*6. CT/MRI WO* + *CEUS late and mild WO*	3/23 (13.0)	17/18 (94.4)	3/4 (75.0)	17/37 (46.0)	20/41 (48.8)
*7. CEUS APHE* + *CT/MRI (capsule enhancement)*	3/23 (13.0)	17/18 (94.4)	3/4 (75.0)	17/37 (46.0)	20/41 (48.8)
*8. CEUS APHE* + *CT/MRI (threshold growth)*	1/23 (4.4)	17/18 (94.4)	1/2 (50.0)	17/39 (46.3)	18/41 (43.9)
*9. CEUS late and mild WO* + *CT/MRI (capsule enhancement)*	1/23 (4.4)	18/18 (100)	1/1 (100)	18/40 (45.0)	19/41 (46.3)
*10. CEUS late and mild WO* + *CT/MRI (threshold growth)*	1/23 (4.4)	16/18 (88.9)	1/3 (33.3)	16/38 (42.1)	17/41 (41.5)
*CT/MRI LR-3* + *LR-4*					
*1. CEUS APHE* + *CT/MRI APHE*	11/30 (36.7)	41/48 (85.4)	11/18 (61.1)	41/60 (68.3)	52/78 (66.7)
*2. CEUS APHE* + *CT/MRI WO*	4/30 (13.3)	47/48 (97.9)	4/5 (80.0)	47/73 (64.4)	51/78 (65.4)
*3. CEUS WO* + *CT/MRI APHE*	3/30 (10.0)	43/48 (89.6)	3/8 (37.5)	43/70 (61.4)	46/78 (59.0)
*4. CEUS WO* + *CT/MRI WO*	3/30 (10.0)	47/48 (97.9)	3/4 (75.0)	47/74 (63.5)	50/78 (64.1)
*5. CT/MRI APHE* + *CEUS late and mild WO*	3/30 (10.0)	45/48 (93.8)	3/6 (50.0)	45/72 (62.5)	48/78 (61.5)
*6. CT/MRI WO* + *CEUS late and mild WO*	3/30 (10.0)	47/48 (97.9)	3/4 (75.0)	47/74 (63.5)	50/78 (64.1)
*7. CEUS APHE* + *CT/MRI (capsule enhancement)*	3/30 (10.0)	47/48 (97.9)	3/4 (75.0)	47/74 (63.5)	50/78 (64.1)
*8. CEUS APHE* + *CT/MRI (threshold growth)*	1/30 (3.3)	47/48 (97.9)	1/2 (50.0)	47/76 (61.8)	48/78 (61.5)
*9. CEUS late and mild WO* + *CT/MRI (capsule enhancement)*	1/30 (3.3)	48/48 (100)	1/1 (100)	48/77 (62.3)	49/78 (62.8)
*10. CEUS late and mild WO* + *CT/MRI (threshold growth)*	1/30 (3.3)	46/48 (95.8)	1/3 (33.3)	46/75 (61.3)	47/78 (60.3)

Data are numerators/denominators. Data in parenthesis are percentages

*APHE* arterial-phase hyper-enhancement, *CEUS* contrast-enhanced ultrasound, *CT* computed tomography, *LI-RADS* Liver Imaging Reporting and Data System, *MRI* magnetic resonance imaging, *NPV* negative predictive value, *PPV* positive predictive value, *WO* washout (regardless of timing or degree)

Among all indeterminate liver observations (Table 3), the most promising combinations were group #7 (CEUS APHE + CT/MRI capsule enhancement) with specificity of 98.5% and PPV of 75.0%, group #6 (CT/MRI WO + CEUS late and mild WO) with specificity of 96.9% and PPV of 71.4%, and group #2 (CEUS APHE + CT/MRI WO) with specificity of 95.4% and PPV of 70.0%. Another potentially helpful combination was group #9 (CEUS late and mild WO + CT/MRI capsule enhancement) with specificity and PPV of 100%. However, for the PPV, there was only one true positive case and no false positive, while there were sixty-five true negative cases for the specificity.

In the CT/MRI LR-M category (Table 3), none of the combined imaging features demonstrated acceptable diagnostic accuracy. The most promising combinations were group #10 (CEUS late and mild WO + CT/MRI threshold growth) with specificity and PPV of 94.1% and 75.0%, and group #6 (CT/MRI WO + CEUS late and mild WO) with specificity and PPV of 94.1% and 66.7%, respectively.

When excluding LR-M and analyzing imaging features combinations for LR-3 + LR-4 liver observations (Table 4), the only potentially helpful combination was group #2 with specificity of 97.9% and PPV of 80.0%. Groups #4 (CEUS WO + CT/MRI WO), #6, and #7 had the same specificities of 97.9% and PPVs 75.0%. For group #9, the specificity and PPV were 100%, but there was only one true positive case and no false positive for PPV.

For LR-4 observations, analysis demonstrated that none of the imaging feature combinations were particularly helpful. Combination groups #4–6 showed similar specificities of 94.4% and PPV of 75.0%. Only groups #2 and #9 showed specificities and PPVs of 100%. However, there was only one true positive case and no false positive case for group #9, while for group #2, there were three true positive cases (Table 4).

**Fig. 4:**
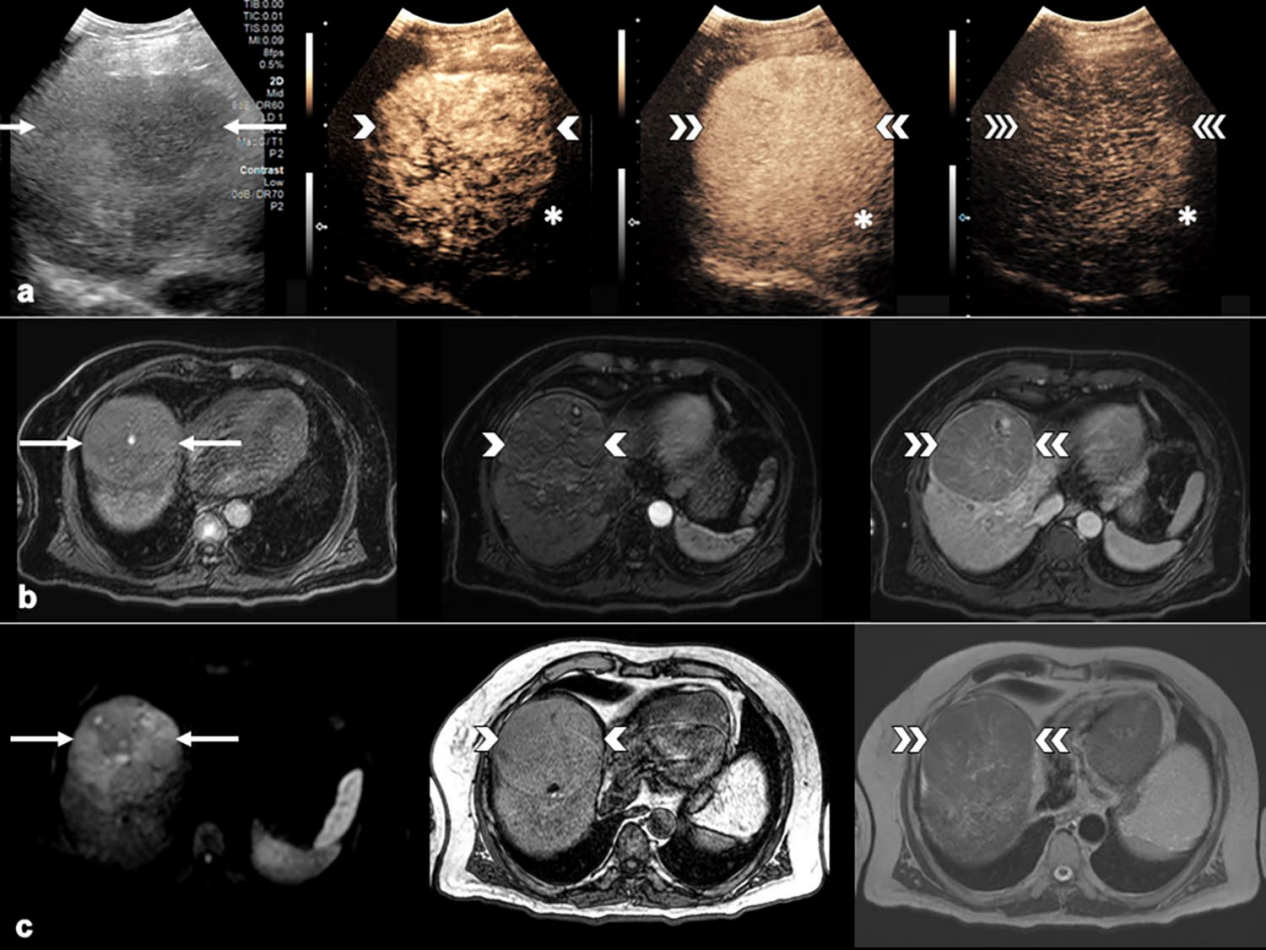
Combination of APHE present on CEUS with WO present on MRI in biopsy-confirmed HCC. **a** B-mode ultrasound demonstrating a large 9.3 cm mass in the right hepatic lobe (arrows). Arterial-phase image demonstrates hyper-enhancement (single arrowhead) with no contrast WO at 2 min (double arrowheads) or 5 min (triple arrowheads), resulting in CEUS LR-4 classification. The contrast agent used was Lumason/SonoVue. **b** Pre-contrast MRI image demonstrated a large mass (arrows) with small amount of T1 hyperintense signal. Arterial-phase image demonstrated iso-enhancement (single arrowhead) with clear WO appearance and capsule enhancement on late-phase image (double arrowheads). **c** Mass also demonstrates restricted diffusion (arrows). Out-of-phase (single arrowhead) images demonstrate intralesional microscopic fat, and T2 image (double arrowheads) demonstrate signal iso-intensity. This combination of image findings resulted in MRI LR-4 classification. APHE, arterial-phase hyper-enhancement; CEUS, contrast-enhanced ultrasound; MRI, magnetic resonance imaging; HCC, hepatocellular carcinoma; WO, washout

**Fig. 5:**
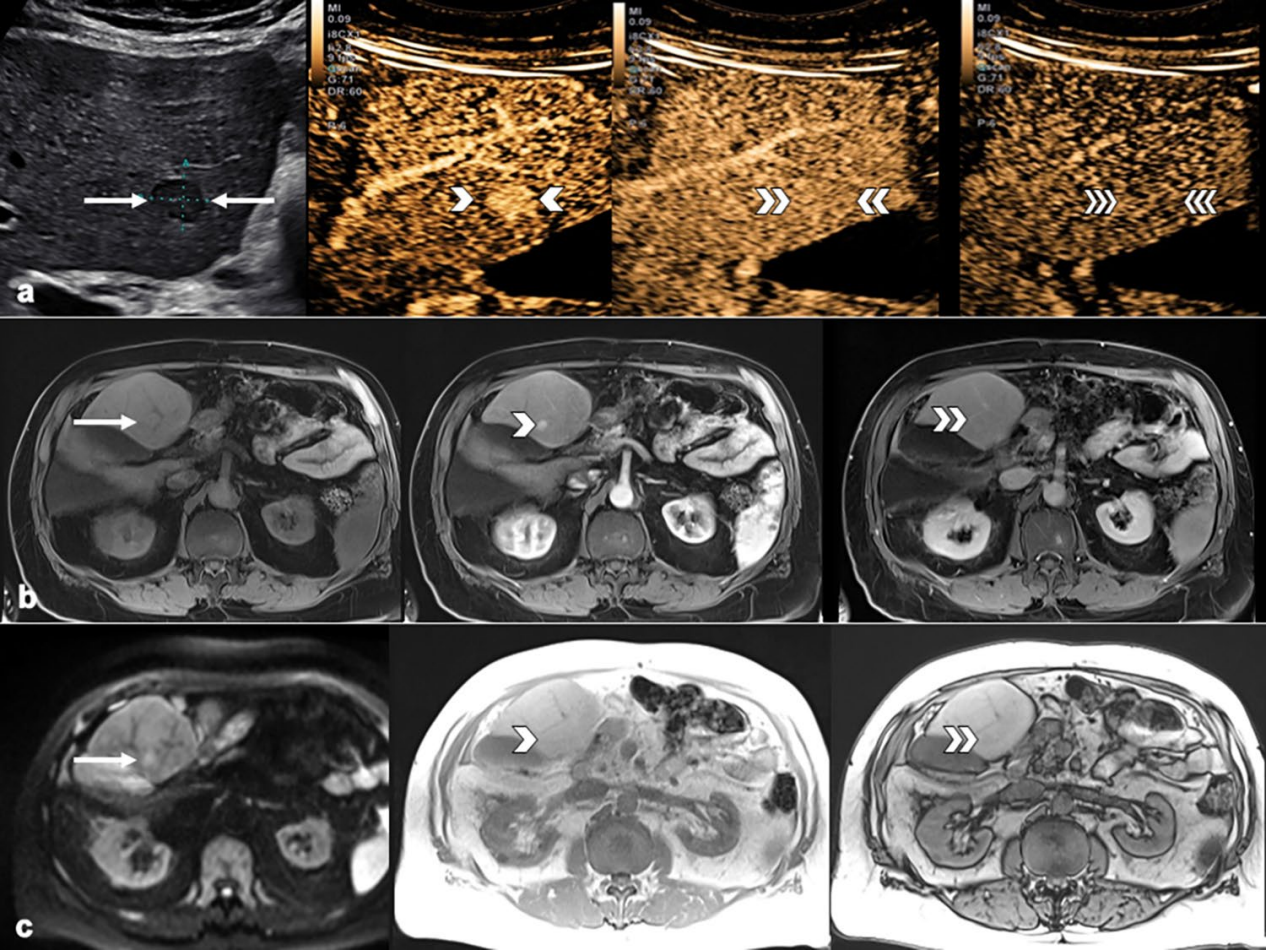
Combination of APHE present on CEUS and MRI in biopsy-confirmed HCC. **a** B-mode ultrasound demonstrating hypoechoic 1.4 cm mass in segment 4B of the left hepatic lobe (arrows). Arterial-phase image demonstrates hyper-enhancement (single arrowhead) with no contrast WO at 1 min (double arrowheads) or 4 min (triple arrowheads), resulting in CEUS LR-4 classification. The contrast agent used was Lumason/SonoVue. **b** Pre-contrast MRI image demonstrated ill-defined observation (arrow). Arterial-phase image demonstrated hyper-enhancement (single arrowhead) without WO on late-phase image (double arrowheads). **c** Observation demonstrates restricted diffusion (arrow). In-phase (single arrowhead) and opposed-phase (double arrowheads) images demonstrate no intralesional fat. This combination of image findings resulted in MRI LR-3 classification. APHE, arterial-phase hyper-enhancement; CEUS, contrast-enhanced ultrasound; MRI, magnetic resonance imaging; HCC, hepatocellular carcinoma; WO, washout

Finally, the highest diagnostic accuracy of imaging features combinations was achieved in CT/MRI LR-3 observations, where group #1 (CEUS APHE + CT/MRI APHE) had 96.7% specificity, 75.0% PPV, 87.9% NPV, and 86.5% accuracy. Additionally, group #2 showed 96.7% specificity, 82.9% NPV, and 81.1% accuracy (Table 4). Examples of these combinations can be seen in Figs. 4 and 5.

A reverse, stepwise logistical regression analysis was used to determine the synergism between the variables corresponding to the major LI-RADS features of each modality, as well as age, and BMI. The *P*-values and A_z_ are shown in Online Resources 6–8.

Among all indeterminate observations between benign and malignant (subgroup 1), CEUS APHE and CT/MRI LI-RADS categories were the independent variables to show statistical significance when assessing for the diagnosis of HCC with *P*-values of 0.005 and < 0.001, respectively, with A_z_ of 0.8914 (Online Resource 7). For HCC versus everything else (subgroup 2), CEUS APHE, CEUS LI-RADS categories, and CT/MRI LI-RADS categories showed *P*-values of 0.0025, 0.0031, and 0.0025, respectively, with A_z_ of 0.7699 (Online Resource 7).

For LR-3 observations in both subgroup 1 and subgroup 2, CEUS APHE was the only independent variable that was statistically significant for assessing indeterminate observations with *P* < 0.0082 and *P* < 0.0057 and A_z_ of 0.7845 and 0.8095, respectively (Online Resource 8).

## Discussion

In this study, we evaluated individual and combined LI-RADS major features from two different algorithms (CEUS LI-RADS v2017 and CT/MRI LI-RADS v2018) to determine whether these combinations could improve the diagnostic performance for the diagnosis of HCC in previously indeterminate liver observations. Importantly, results demonstrated that a combination of individual imaging features in cases with indeterminate categorization on both CT/MRI and CEUS, can achieve a final diagnosis of HCC, especially in CT/MRI LR-3 observations.

CT/MRI and CEUS LI-RADS major features, such as APHE, WO, capsule enhancement, and threshold growth, have been assessed independently in previous studies [9, 17, 18]. In a systematic review and meta-analysis study, van der Pol et al. evaluated every LI-RADS major feature on CEUS and CT/MRI to determine the probability of HCC. According to the study, APHE and WO were two individual major features strongly associated with HCC in both modalities, while threshold growth showed no association [17]. Capsule enhancement on CT/MRI and late and mild WO on CEUS also displayed an association with HCC [17]. Similar to our study, CEUS APHE was a variable strongly associated with HCC. Although this variable was the only strong association, this can be due to the sample size difference and study design as compared to van der Pol et al. However, the study only evaluated LI-RADS major features individually unlike our current study. Alternatively, in a retrospective study by Choi et al., the threshold growth in the diagnosis of HCC was evaluated, which was highly associated with HCC and indicated that using threshold growth can enhance the sensitivity of LI-RADS v2018 [18]. Nonetheless, the study used only threshold growth as LI-RADS major features, and the number of observations was lower compared to van der Pol et al. According to these two previous studies, all LI-RADS major features individually showed high association with HCC [17, 18].

Our study showed that the diagnostic performance of HCC improved with combined LI-RADS major features on CEUS and CT/MRI when compared to individual major features in cases with indeterminate results. Furthermore, for every LI-RADS category in the present study, the most helpful and promising combinations were seen when APHE, WO, and capsule enhancement on CT/MRI, and APHE on CEUS, were part of the combination. These results are comparable to previous studies by Caraiani et al., where the CEUS APHE showed high specificity and PPV, and by Shin et al., where the performance of MRI LI-RADS major features, including APHE, WO, and enhancing capsule were close to our study [9, 19]. Caraiani et al. also suggested the use of both CT/MRI and CEUS LI-RADS to assess indeterminate liver observations [19].

Multiple studies compare the efficacy of CEUS and CT/MRI to reclassify liver observations [20–22]. However, to our knowledge, there is no published data about the combination of these modalities and LI-RADS major features and their clinical impact on the diagnosis of HCC in patients with indeterminate observations. Zhou et al. combined CEUS with CT/MRI to reclassify inconclusive liver observations to improve the diagnostic performance for HCC. CEUS was used to upgrade 40 LR-3 and LR-4 liver observations identified by CT/MRI, from which 35 were found to be HCC [23]. Based on CEUS major features, 13/40 liver observations were upgraded due to APHE, 23/40 by WO, 2/40 by both APHE and WO, and 2/40 by size. The same categorization of LR-M for 6 observations in CT/MRI was obtained by CEUS. The study concluded that a definitive diagnosis of HCC can be obtained using CEUS to reclassify liver observations in CT/MRI LR-3 and LR-4 [23]. However, the study used CEUS LI-RADS major features to reclassify rather than combine features across two imaging modalities. A 2017 study by Beyer et al. concluded that CEUS and MRI are equally effective imaging tests to detect and characterize focal liver observations and that false-negative cases can be reduced by combining these modalities [24]. However, the study combined these two imaging modalities with clinical data, available biopsy, and changes in size or appearance in a follow-up imaging test. Furthermore, specific LI-RADS major features were not used in the study [24].

Our study has some limitations. First, it is important to acknowledge that our study is retrospective, and we performed data analyses according to the information collected from the original prospective study. Second, while we meticulously excluded cases based on specific selection criteria, this process inevitably led to a reduction in sample size, thereby limiting the generalizability of our findings. Indeed, the sample size in our study does not adequately represent the global population. In addition, we performed analyses on combinations of only 2 dominant imaging features due to the small sample size. For this reason, multiple feature combinations should be addressed in a larger, prospective, multicenter study.

We showed that the combination of CEUS APHE + CT/MRI APHE had higher specificity, PPV, and accuracy for diagnosing HCC compared to the individual modalities alone, especially in observations originally categorized as CT/MRI LR-3.

## Supplementary Information

Below is the link to the electronic supplementary material.

Supplementary file1 (PDF 274 KB)

## Data Availability

No datasets were generated or analysed during the current study.

## Abbreviations

A_z_: Area under the receiver operating characteristic curve
APHE: Arterial phase hyper-enhancement
CEUS: Contrast-enhanced ultrasound
HCC: Hepatocellular carcinoma
LI-RADS: Liver imaging reporting and data system
NPV: Negative predictive value
PPV: Positive predictive value
WO: Washout
