# Supplementary material for: Combining CEUS and CT/MRI LI-RADS major imaging features: diagnostic accuracy for classification of indeterminate liver observations in patients at risk for HCC
Source: Abdom Radiol (NY). 2024 Oct 23;50(5):2066–77. doi: 10.1007/s00261-024-04625-w (PMC11991985; doi:10.1007/s00261-024-04625-w)
Supplement: Supplementary file 1 — Supplementary file1 (PDF 274 KB) [file 261_2024_4625_MOESM1_ESM.pdf]

Combining CEUS and CT/MRI LI-RADS Major Imaging Features: Diagnostic Accuracy for Classification of Indeterminate Liver Observations in Patients at Risk for HCC

Journal Name: Abdominal Radiology Journal

Tania Siu Xiao, MD[1]; Cristina Mariuxi Kuon Yeng Escalante, MD[1]; Aylin Tahmasebi, MD[1]; Yuko Kono, MD, PhD, FAIUM, FAASLD[2]; Fabio Piscaglia, MD, PhD[3,4]; Stephanie R. Wilson, MD, FRCPC[5]; Alexandra Medellin-Kowalewski, MD, FRCPC[5]; Shuchi K. Rodgers, MD[1,6]; Virginia Planz, MD[7]; Aya Kamaya, MD[8]; David T. Fetzer, MD[9]; Annalisa Berzigotti, MD, PhD[10]; Iuliana-Pompilia Radu, MD[10]; Paul S. Sidhu, BSc, MBBS, MRCP, FRCR, DTM&H, FCIRSE, FAIUM[11]; Corinne E. Wessner, MS, MBA, RDMS, RVT[1]; Kristen Bradigan, RN, BSN[1]; John R. Eisenbrey, PhD[1]; Flemming Forsberg, PhD, FAIUM, FAIMBE[1]; Andrej Lyshchik, MD, PhD[1]; CEUS LI-RADS Trial Group.

[1] Thomas Jefferson University Hospital, Philadelphia PA, United States
[2] University of California, San Diego, San Diego CA, United States
[3] Division of Internal Medicine, Hepatobiliary and Immunoallergic Diseases, IRCCS Azienda Ospedaliero-Universitaria di Bologna, Italy
[4] Department of Medical and Surgical Sciences, University of Bologna, Italy
[5] University of Calgary, Calgary, Canada
[6] Einstein Medical Center, Philadelphia PA, United States
[7] Vanderbilt University, Nashville TN, United States
[8] Stanford University, Stanford CA, United States
[9] UT Southwestern Medical Center, Dallas, TX, United States
[10] Department of Visceral Surgery and Medicine, Bern University Hospital, University of Bern, Bern, Switzerland
[11] Department of Imaging Sciences, School of Biomedical Engineering and Imaging Sciences, Faculty of Life Sciences and Medicine, King's College London. Department of Radiology, King's College Hospital, London, UK

Corresponding Author:
Andrej Lyshchik
Associate Professor of Radiology
andrej.lyshchik@jefferson.edu

SUPPLEMENTAL TABLES AND FIGURES

Online Resource 1 Combination groups of multimodality imaging with LI-RADS imaging major features

Groups	*Multimodality Combinations of LI-RADS Major Features*
#1	CEUS APHE + CT/MRI APHE
#2	CEUS APHE + CT/MRI WO
#3	CEUS WO + CT/MRI APHE
#4	CEUS WO + CT/MRI WO
#5	CT/MRI APHE + CEUS late and mild WO
#6	CT/MRI WO + CEUS late and mild WO
#7	CEUS APHE + CT/MRI (capsule enhancement)
#8	CEUS APHE + CT/MRI (threshold growth)
#9	CEUS late and mild WO + CT/MRI (capsule enhancement)
#10	CEUS late and mild WO + CT/MRI (threshold growth)

APHE = arterial-phase hyper-enhancement, CEUS = contrast-enhanced ultrasound, CT = computed tomography, LI-RADS = Liver Imaging Reporting and Data System, MRI = magnetic resonance imaging, WO = washout (regardless of timing or degree)

Online Resource 2 Variables used for the stepwise logistical regression analysis

VARIABLES	***DESCRIPTION***
Dependent	
Pathology (Subgroup 1)	Benign or all malignancy (HCC and non-HCC malignancy)
Pathology (Subgroup 2)	HCC or everything else (benign and non-HCC malignancy)
Independent	
CEUS APHE	Hypo-enhancement, iso-enhancement, rim-enhancement, or hyperenhancement
CEUS WO (Presence)	Absent or present
CEUS WO (Degree)	Mild or marked
CEUS WO (Time)	Early or late
CEUS WO (Time + Degree)	Early, late + marked, or late + mild
CT/MRI APHE	Hypo-enhancement, iso-enhancement, rim-enhancement, hyperenhancement, or peripheral discontinuous enhancement
CT/MRI WO	Absent or present
CT/MRI capsule enhancement	Absent or present
CT/MRI threshold growth	Absent or present
CEUS LI-RADS categories	LR-3, LR-4, LR-M, or LR-TIV
CT/MRI LI-RADS categories	LR-3, LR-4, or LR-M
Age	Original value
BMI	Original value

APHE = arterial-phase hyper-enhancement, BMI = body mass index, CEUS = contrast-enhanced ultrasound, CT = computed tomography, HCC = hepatocellular carcinoma, LI-RADS = Liver Imaging Reporting and Data System, MRI = magnetic resonance imaging, WO = washout

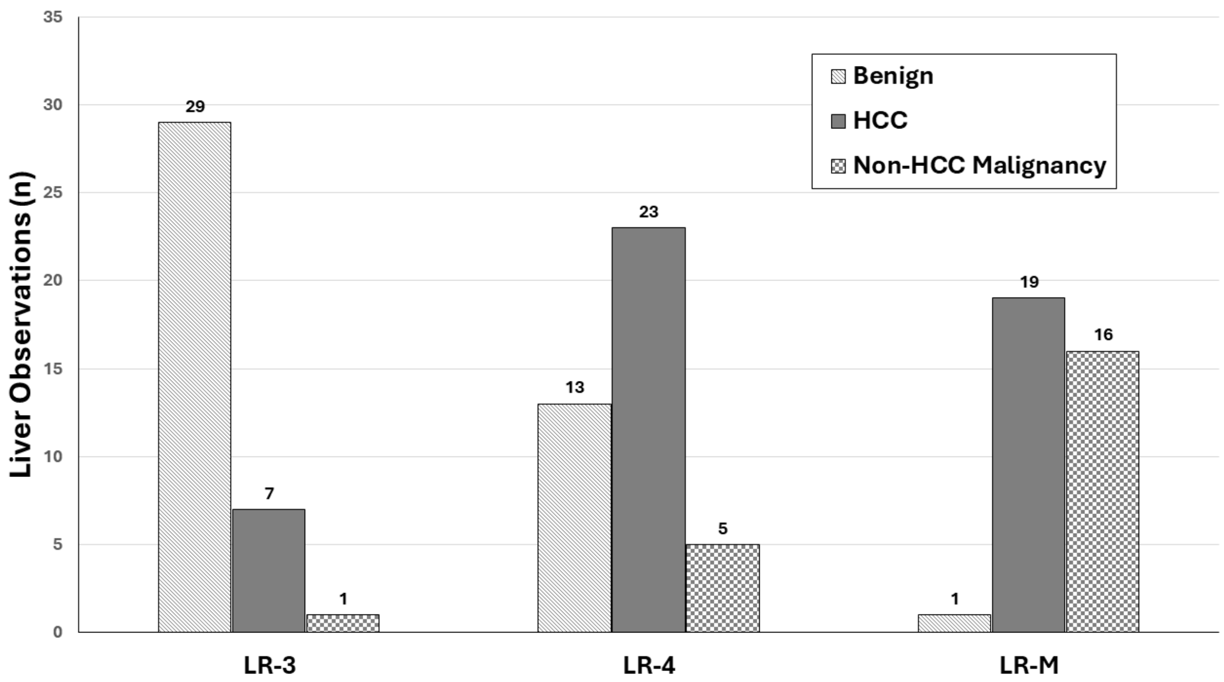

Online Resource 3 Final diagnosis of initial CT/MRI indeterminate liver observations

HCC = hepatocellular carcinoma

Online Resource 4 Initial CEUS and CT/MRI LI-RADS classification of liver observations with the final diagnosis

Final Diagnosis		***Initial CEUS LI-RADS***	***Initial CT/MRI LI-RADS***	***Total cases (n)***
Benign				
Follow-up CT/MRI	LR-3	28	27	35
	LR-4	6	8	
	LR-M	1	0	
Biopsy	LR-3	0	0	5
	LR-4	5	4	
	LR-M	0	1	
Explant Histology	LR-3	3	2	3
	LR-4	0	1	
	LR-M	0	0	
HCC				
Follow-up CT/MRI	LR-3	6	3	17
	LR-4	10	11	
	LR-M	1	3	
Biopsy	LR-3	5	3	23
	LR-4	11	6	
	LR-M	6	14	
	LR-TIV	1	0	
Explant Histology	LR-3	5	1	9
	LR-4	2	6	
	LR-M	2	2	
Other malignancy				
Follow-up CT/MRI	LR-3	0	0	1
	LR-4	0	0	
	LR-M	1	1	
Biopsy	LR-3	0	0	20
	LR-4	2	5	
	LR-M	18	15	
Explant Histology	LR-3	1	1	1
	LR-4	0	0	
	LR-M	0	0	

CEUS = contrast-enhanced ultrasound, CT = computed tomography, HCC = hepatocellular carcinoma, LI-RADS = Liver Imaging Reporting and Data System, MRI = magnetic resonance imaging

Online Resource 5 Distribution of CEUS and CT/MRI LI-RADS major features for all indeterminate liver nodules

CEUS* LI-RADS Major Features		***CT/MRI LI-RADS Major Features***	
**CEUS LR-3**	**n**	**CT/MRI LR-3**	**n**
Total Nodules	48	Total Nodules	37
APHE		***APHE***	
• Hyperenhancement	1	• Hyperenhancement	16
• Isoenhancement	32	• Isoenhancement	13
• Hypoenhancement	15	• Hypoenhancement	7
• Rim-enhancement	0	• Rim-enhancement	0
		• No Enhancement	1
WO		***WO***	
• Present	3	• Present	8
• Absent	45	• Absent	29
WO Timing		***Capsule Enhancement***	
• Early	0	• Present	0
• Late	3	• Absent	37
WO Degree		***Threshold Growth***	
• Mild	3	• Present	0
• Marked	0	• Absent	37
**CEUS LR-4**	**n**	**CT/MRI LR-4**	**n**
Total Nodules	36	Total Nodules	41
APHE		***APHE***	
• Hyperenhancement	26	• Hyperenhancement	27
• Isoenhancement	5	• Isoenhancement	11
• Hypoenhancement	5	• Hypoenhancement	2
• Rim-enhancement	0	• Rim-enhancement	0
		• Peripheral Discontinuous Enhancement	1
WO		***WO***	
• Present	9	• Present	13
• Absent	27	• Absent	28
Wo Timing		***Capsule Enhancement***	
• Early	0	• Present	6
• Late	9	• Absent	35
Wo Degree		***Threshold Growth***	
• Mild	9	• Present	5

• Marked	0	• Absent	36
**CEUS LR-M**	**n**	**CT/MRI LR-M**	**n**
Total Nodules	29	Total Nodules	36
APHE		***APHE***	
• Hyperenhancement	12	• Hyperenhancement	9
• Isoenhancement	2	• Isoenhancement	3
• Hypoenhancement	2	• Hypoenhancement	0
• Rim-Enhancement	13	• Rim-enhancement	24
WO		***WO***	
• Present	28	• Present	10
• Absent	1	• Absent	26
WO Timing		***Capsule Enhancement***	
• Early	13	• Present	3
• Late	15	• Absent	33
WO Degree		***Threshold Growth***	
• Mild	12	• Present	10
• Marked	16	• Absent	26

Note. *CEUS LI-RADS classified one nodule as LR-TIV (isoenhancement, WO present, late WO, and mild WO). For this case, CT/MRI LI-RADS classified it as LR-3 (Hypoenhancement, WO absent, no capsule enhancement, and no threshold growth).

APHE = arterial-phase hyper-enhancement, CEUS = contrast-enhanced ultrasound, CT = computed tomography, LI-RADS = Liver Imaging Reporting and Data System, MRI = magnetic resonance imaging, WO = washout

Online Resource 6 Results of stepwise logistical regression analysis

CATEGORIES	***P-value***	***ROC***
All CT/MRI observations		
Benign vs All Malignant		
o *CEUS APHE*	0.005**	0.8914
o *CT/MRI LI-RADS*	<0.001***	0.8914
HCC vs Everything Else		
o *CEUS APHE*	0.0025**	0.7699
o *CEUS LI-RADS*	0.0031**	0.7699
o *CT/MRI LI-RADS*	0.0025**	0.7699
CT/MRI LR-3		
Benign vs All Malignant		
o *CEUS APHE*	0.0082**	0.7845
HCC vs Everything Else		
o *CEUS APHE*	0.0057**	0.8095
CT/MRI LR-4		
Benign vs All Malignant		
o *CEUS APHE*	0.2875 (ns)	0.5989
HCC vs Everything Else		
o *Age*	0.0288*	0.6739
CT/MRI LR-M		
Benign vs All Malignant		
o *CT/MRI APHE*	0.7598 (ns)	0.5857
HCC vs Everything Else		
o *CEUS LI-RADS*	0.0149*	0.7167
CT/MRI LR-3 + LR-4		
Benign vs All Malignant		
o *CEUS APHE*	0. 0144*	0.8016
o *CT/MRI LI-RADS*	0.0004***	0.8016
HCC vs Everything Else		
o *Age*	0.0367*	0.7899
o *CEUS APHE*	0.0113*	0.7899
o *CT/MRI LI-RADS*	0.0025**	0.7899

Note. —ns (nonsignificant) P > 0.05, *P ≤ 0.05, **P ≤ 0.01, ***P ≤ 0.001

APHE = arterial-phase hyper-enhancement, BMI = body mass index, CEUS = contrast-enhanced ultrasound, CT = computed tomography, CI = confidence interval, HCC = hepatocellular carcinoma, LI-RADS = Liver Imaging Reporting and Data System, MRI = magnetic resonance imaging, ROC = Receiver operating characteristic

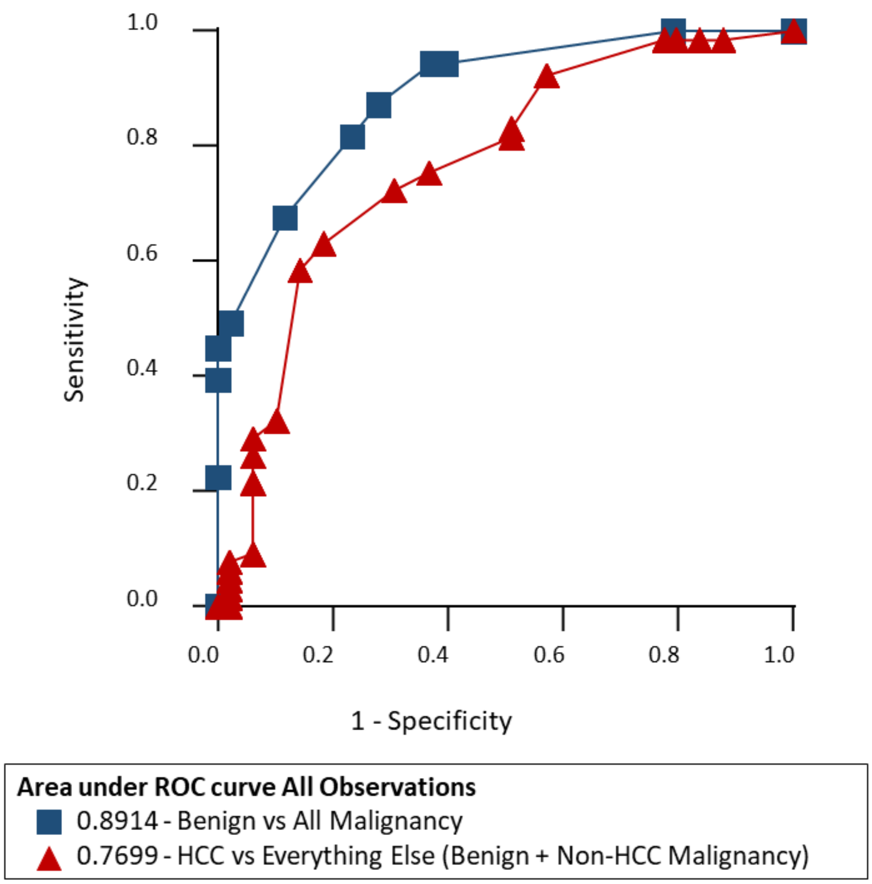

Online Resource 7 Area under ROC curve for all observations

HCC = Hepatocellular carcinoma, ROC = Receiver operating characteristic

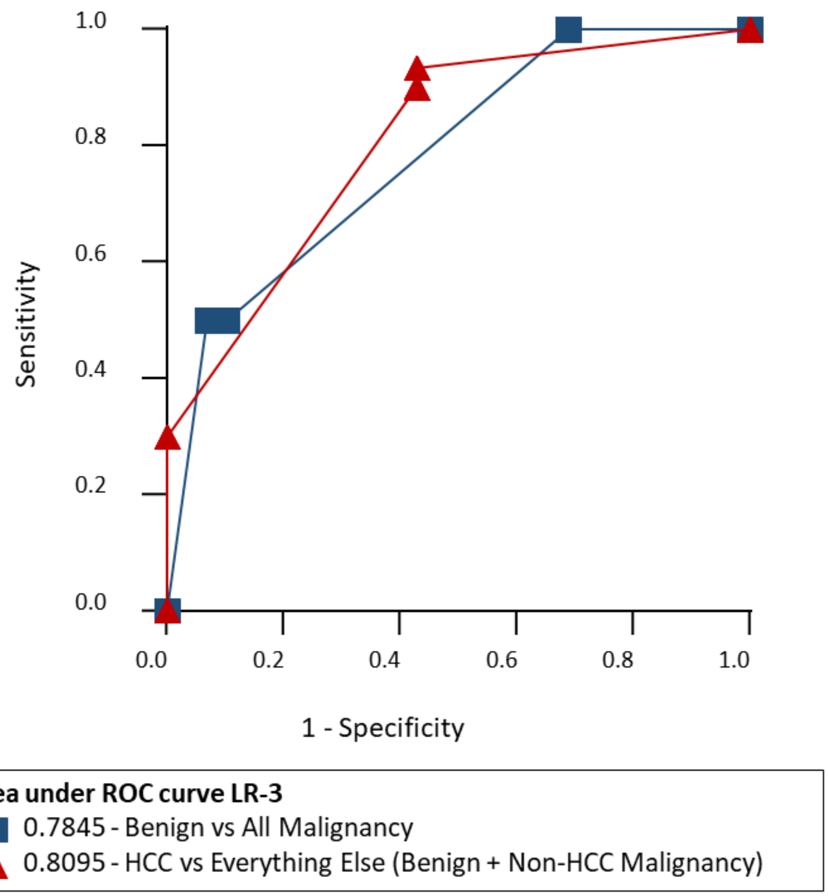

Online Resource 8 Area under ROC curve for LR-3 observations

HCC = Hepatocellular carcinoma, ROC = Receiver operating characteristic
